# Ubiquitous mitochondrial creatine kinase downregulated in oral squamous cell carcinoma

**DOI:** 10.1038/sj.bjc.6602986

**Published:** 2006-02-14

**Authors:** T Onda, K Uzawa, Y Endo, H Bukawa, H Yokoe, T Shibahara, H Tanzawa

**Affiliations:** 1 Department of Oral and Maxillo-Facial Surgery, Tokyo Dental College, 1-2-2 Masago, Mihama-ku Chiba 261-8502, Japan; 2 Department of Clinical Molecular Biology, Graduate School of Medicine, Chiba University, 1-8-1 Inohana, Chuo-ku Chiba 260-8670, Japan; 3 Division of Dentistry and Oral-Maxillofacial Surgery, Chiba University Hospital, 1-8-1 Inohana, Chuo-ku Chiba 260-8677, Japan; 4 The 21st Century Center of Excellence (COE) program, Graduate School of Medicine, Chiba University, 1-8-1 Inohana, Chuo-ku Chiba 260-8670, Japan

**Keywords:** CKMT1, oral squamous cell carcinoma, two-dimensional electrophoresis, MALDI-TOF-mass spectrometry, transfection

## Abstract

In this study, we performed two-dimensional electrophoresis (2-DE) and matrix-assisted laser desorption/ionisation time of fly mass spectrometry to identify the protein(s) associated with the development of oral squamous cell carcinomas (OSCCs) by comparing patterns of OSCC-derived cell lines with normal oral keratinocytes (NOKs), and found that downregulation of ubiquitous mitochondrial creatine kinase (CKMT1) could be a good candidate. Decreased levels of CKMT1 mRNA and protein were detected in all OSCC-derived cell lines examined (*n*=9) when compared to those in primary normal oral keratinocytes. Although no sequence variation in the coding region of the *CKMT1* gene with the exception of a nonsense mutation in exon 8 was identified in these cell lines, we found a frequent hypermethylation in the CpG island region. *CKMT1* expression was restored by experimental demethylation. In addition, when we transfected CKMT1 into the cell lines, they showed an apoptotic phenotype but no invasiveness. In clinical samples, high frequencies of CKMT1 downregulation were detected by immunohistochemistry (19 of 52 (37%)) and quantitative real-time RT–PCR (21 of 50 (42%)). Furthermore, the CKMT1 expression status was significantly correlated with tumour differentiation (*P*<0.0001). These results suggest that the *CKMT1* gene is frequently inactivated during oral carcinogenesis and that an epigenetic mechanism may regulate loss of expression, which may lead to block apoptosis.

Oral cancer is a challenging clinical problem and a leading cause of cancer deaths for both men and women, with an estimated 300 000 new cases annually worldwide (Greenlee *et al*, 2000). Many risk factors have already been identified, including human papilloma virus infection, tobacco use, and alcohol consumption. However, some patients develop oral squamous cell carcinoma (OSCC) without risk factors, suggesting that host susceptibility may play a role. Molecular alterations in a number of oncogenes and tumour-suppressor genes associated with the development of OSCC could be important clues to preventing this disease.

Gene expression profiles using cDNA microarrays have been used to derive a useful molecular-based classification for the diagnosis, treatment, and prevention of several cancers (Alizadeh *et al*, 2000; Bittner *et al*, 2000). However, biologic systems comprise protein components resulting from transcriptional and post-transcriptional control, post-translational modifications, and protein shifts among different cellular compartments. These properties cannot be analysed by microarray systems at the RNA level, whereas proteomic analysis allows separation and visualisation of the protein content of a cellular sample (Celis *et al*, 1996). Considering this, proteome analysis may be a more powerful analytic technology to enhance study of the diagnosis, treatment, and prevention of human diseases, including human cancers (Sanchez *et al*, 1997; Wolfsberg *et al*, 2002).

Two-dimensional electrophoresis (2-DE) is the principal tool in proteomics, which can analyse thousands of proteins in one experiment, providing the highest resolution in protein separation. By comprehensively examining the protein expression profiles in normal and diseased samples via 2-DE, proteomics provides information on new biomarkers, disease-associated targets, and pathogenesis (Alaiya *et al*, 2000; Chambers *et al*, 2000; Miklos and Maleszka, 2001). This technique has been used extensively to investigate various diseases, including SCC of the oesophagus (Zhang *et al*, 2003), bladder (Ostergaard *et al*, 1997; Celis *et al*, 1999, 2000), lung (Li *et al*, 2003a, 2003b, 2004), and head and neck (Wu *et al*, 2002; Drake *et al*, 2005; Roesch Ely *et al*, 2005). However, little is known about the state of protein expression profiles in OSCCs. The aim of the present study was to identify the protein(s) associated with the development of OSCCs by comparing the patterns of OSCC-derived cell lines with normal oral keratinocytes (NOKs) using 2-DE and matrix-assisted laser desorption/ionisation time of fly mass spectrometry (MALDI-TOF-mass). We also examined the genetic and epigenetic changes and performed functional analysis of the candidate gene product identified.

## MATERIALS AND METHODS

### Cells

The nine human OSCCs-derived cell lines used in this study were Ca9-22, Ho-1-N-1, HSC-2, HSC-3, HSC-4, SAS (Human Science Research Resources Bank, Osaka, Japan), OK92 (established from carcinoma of the tongue in our department), Sa3, and H1 (provided by Dr Shigeyuki Fujita, Wakayama Medical University, Wakayama, Japan). All cell lines were maintained at 37°C (humidified atmosphere 5% CO_2_/95% air) on 150 × 20-mm tissue culture dishes (Nunc, Roskilde, Denmark) and cultured in Dulbecco's modified Eagle's medium F-12 HAM (Sigma Chemical Co, St Louis, MO, USA) with 10% fetal bovine serum (Sigma) and 50 U ml^−1^ penicillin and streptomycin. Primary cultured NOKs were used as a normal control as described previously (Kang *et al*, 2000; Endo *et al*, 2004).

### Tissue samples and nucleic acid isolation

Tumours with patient-matched normal oral tissues (when available) were obtained at the time of surgical resection at Chiba University Hospital after informed consent had been obtained from the patients according to a protocol that was approved by the institutional review board of Chiba University. Resected tissues were divided into two parts: one was frozen immediately and stored at −80°C until use, and the other was fixed in 10% buffered formaldehyde solution for pathologic diagnosis. Histopathologic diagnosis of each cancerous tissue was performed according to the International Histological Classification of Tumours (Wahi, 1971) by the Department of Pathology, Chiba University Hospital. Clinicopathologic staging was determined by the TNM Classification of the International Union against Cancer (Hermanek *et al*, 1987). All patients had OSCC that was histologically confirmed, and tumour samples were checked to ensure the presence of tumour tissue in more than 80% of specimens. Genomic DNA was extracted as described previously (Uzawa *et al*, 1994); total RNA was prepared using Trizol Reagent (Invitrogen Corp., Carlsbad, CA, USA), according to the manufacturer's protocol.

### Two-dimensional polyacrylamide gel electrophoresis

The protein expression profiles of seven OSCCs-derived cell lines (Ca9-22, OK-92, Ho1-N-1, HSC4, SAS, Sa3, and H1) and four NOKs were examined (Figure 2A). When the cultured cells grew into a full monolayer, they were washed three times with cold PBS (Sigma) and treated with lysis buffer containing 30 mM Tris-HCl (pH 7.5), 150 mM NaCl, 1% Triton X-100, 10% glycerol, and protease inhibitor cocktail to generate protein lysates for 10 min at 4°C. The cells were collected with a scraper and centrifuged at 15 000 r.p.m. for 10 min at 4°C. Protein concentrations were determined by the Bradford method. Total protein concentrations were normalised to 1 *μ*g *μ*l^−1^ for all samples, and the sample aliquots were stored at −20°C until use.

For 2-D polyacrylamide gel electrophoresis (PAGE), each protein samples (100 *μ*g) was mixed with sample buffer containing 7 M urea, 2 M thiourea, 4% (w v^−1^) 3-((3-cholamidopropyl)dimethylammonio)-1-propanesulphonic acid (CHAPS), and 1% (w v^−1^) DTT. Isoelectric focusing for the first dimension of protein separation was performed with a Multiphore II electrophoresis system (Amersham Biosciences, Piscataway, NJ, USA). Immobiline Dry IPG strips (7 cm, pH 3–10 nonlinear IPG strips; Amersham Biosciences) were rehydrated overnight with the sample/rehydration buffer mixture. The strips were subjected to electrophoresis using a ramping IPG strip (200–5000 V) focusing algorithm. After isoelectric focusing, the gel strips were equilibrated with sodium dodecyl sulohate (SDS) equilibration buffer and electrophoresed in vertical SDS–PAGE slab gels containing 12.5% acrylamide. The gels of the microdissected samples were fixed and silver stained using a Silver Quest silver staining kit (Invitrogen Japan, K.K., Tokyo, Japan), according to the manufacturer's instructions.

### Two-dimensional gel analysis and MALDI-TOF-mass spectrometry

The silver stained gel was scanned into Adobe Photoshop 4.0J. (Adobe, San, Jose, CA, USA) with a Umax Powerlook II scanner (Umax, Dallas) and printed. Differences in protein levels were defined as clear visual differences in size, density, or both of the protein spot on the gel. Phoretix 2D Advanced software (Version 5.01, Nonlinear Dynamic, Ltd., Newcastle, UK) was used to estimate the relative differences in spot intensity of a candidate protein. Spots that were consistently and substantially different were selected for MALDI-TOF-mass analysis (Figure 2A). The protein spots were excised from the gels, and in-gel digestion was performed with an enzyme solution containing 50 mM NH_4_HCO_3_, 5 mM CaCl_2_, and 12.5 ng *μ*l^−1^ trypsin. Aliquots of the purified samples were spotted on matrix crystals of *α*-cyano-4-hydroxyl-cinnamic acid on a stainless steel target and air dried. Mass determinations were performed on the AXIMA-CFR mass spectrometer (Shimadzu Co. Ltd, Kyoto, Japan). The proteins were identified by the peptide-mass fingerprinting method using Mascot Search on the Web (Matrix Science, Ltd, London, UK).

### Immunofluorescence

Cells were grown on glass coverslips, fixed with methanol for 5 min, and permeabilised in 0.01%. Triton X-100 in PBS (pH 7.4) for 10 min. Nonspecific binding was blocked with 5% skim milk in PBS (pH 7.4) for 1 h. Cells were further incubated for 2 h with goat anti-CKMT1 (Santa Cruz Biotechnology, Santa Cruz, CA, USA) at a dilution of 1:100, washed with PBS, and incubated with rabbit anti-goat secondary antibody labelled with Alexa Fluor 488 (Molecular Probes, Leiden, the Netherlands) for 1 h. Further, a DAPI nuclear stain with (4,6-diamidino-2-phenylindole dihydrochloride) was performed (0.5 *μ*g ml^−1^). Coverslips were examined by fluorescence microscopy (Figure 2A). The microscope used was a Leica DMIRBE inverted stand equipped with a Leica TCS2-MP confocal system (Leica Laserteknik, Mannheim, Germany) and Coherent Mira tunable pulsed titanium sapphire laser (Coherent Laser Group, Santa Clara, CA, USA).

### Immunohistochemistry

Immunohistochemistry (IHC) staining was performed on 4-*μ*m paraffin-embedded specimens (Figure 2A). Briefly, after deparaffinisation and hydration, slides were treated with endogenous peroxidase in 0.3% H_2_O_2_ for 30 min and the sections blocked for 2 h at room temperature with 1.5% blocking serum (Santa Cruz Biotechnology) in PBS before reacting with anti-CKMT1 (Santa Cruz Biotechnology) at a dilution of 1 : 500. Sections then were incubated with primary antibody for 30 min at room temperature in a moist chamber. After incubation, the sections were washed three times in PBS buffer and treated with Envision reagent (Dako, Kyoto, Japan), followed by colour development in 3,3′-diaminobenzidine tetrahydrochloride (Dako). Finally, slides were lightly counterstained with haematoxylin and mounted. As a negative control, duplicate sections were immunostained without exposure to primary antibodies. To quantitate CKMT1 protein expression, a scoring method was used in which the mean percentage of positive tumour cells was determined in at least five random fields at × 400 magnification in each section. The intensity of the CKMT1 immunoreaction was scored as follows: 1+, weak; 2+, moderate; and 3+, intense. The percentage of positive tumour cells and the staining intensity were multiplied to produce a CKMT1 IHC staining (CKMT1-IHC) score for each case. Cases with a CKMT1-IHC score less than 62.2 (minimum score of normal tissues) were considered negative. Two independent pathologists without knowledge of or information pertaining to the patients' clinical status scored the cases. A statistically significance difference between the CKMT1-IHC scores and clinicopathologic features was evaluated by the Mann–Whitney's *U*-test. *P*<0.05 was considered significant.

### mRNA expression analysis

The expression levels of *CKMT1* mRNA were examined in the tumours, and paired normal oral tissues from the 50 patients with OSCC, nine OSCC-derived cell lines, and NOKs were analysed (Figure 2A). Control reactions were prepared in parallel without reverse transcriptase. Before cDNA synthesis, residual genomic DNA was removed from the total RNA by DNase I treatment (DNA-free; Ambion, Austin, TX, USA). The primer sequences used for analysis of *CKMT1* mRNA expression were 5′-CCTGCTAAGCAAAGATAGCC-3′ (nucleotides 1162–1181), 5′-TAATGCTTGGTGTGGATGAC-3′ (nucleotides 1397–1416). The sequence of specific primers was checked before use to avoid amplification of genomic DNA or pseudogenes using the Primer3 program (http://www-genome.wi.mit.edu/cgibin/primer/primer3_www.cgi). The amplified products were analysed by 3% agarose gel electrophoresis to ascertain the size and purity of the products. To confirm the identity of the PCR products, they were cloned into a pCR 2.1vector (Invitrogen) and sequenced as described previously (Endo *et al*, 2004).

Real-time quantitative RT–PCR was performed with a single method using a LightCycler FastStart DNA Master SYBR Green I kit (Roche Diagnostics GmbH, Mannheim, Germany). To prepare the standard curve, 1.5 *μ*g of total RNA from normal oral tissue was reverse transcribed with Superscript reverse transcriptase (Life Technologies, Grand Island, NY, USA) and oligod (T) primer, after which serial dilutions were made corresponding to cDNA transcribed from 300, 30, 3.0, and 0.3 ng of total RNA. The PCR reactions using the LightCycler (Roche Diagnostics) apparatus were carried out in a final volume of 20 *μ*1 of reaction mixture consisting of 2 *μ*1 of FirstStart DNA Master SYBR Green I mix (Roche), and 3 mM MgCl_2_, 0.2 *μ*1 of the primers, according to the manufacturer's instructions. The reaction mixture was loaded into glass capillary tubes and submitted to an initial denaturation at 95°C for 10 min, followed by 45 rounds of amplification at 95°C (10 s) for denaturation, 56°C (10 s) for annealing, and 72°C for extension, with a temperature slope of 20°C s^−1^, performed in the LightCycler. The transcript amount for the *CKMT1* gene was estimated from the respective standard curves and normalised to the amount of glyceraldehyde-3-phosphate dehydrogenase (GAPDH) transcript determined in corresponding samples.

### Mutational analyses

To screen the sequence variations of the *CKMT1* gene, PCR-single-strand confirmation polymorphism (SSCP) analysis and DNA sequencing analysis were performed as described previously (Uzawa *et al*, 1995; Endo *et al*, 2004) (Figure 2A). Nine sets of oligonucleotide primers as summarised in Table 3 were used to amplify the entire coding region (exons 1–9) of the *CKMT1* gene. Sequences for all annotated exons and adjacent intronic sequences were extracted from public (http://www.ncbi.nlm.nih.gov/) draft human genome sequences. Primers for PCR amplification and sequencing were designed using the Primer 3 program as described above.

### Methylation analyses

To determine if methylation of a CpG island of the *CKMT1* gene contributes to the mRNA expression of *CKMT1*, DNA samples obtained from the nine OSCC-derived cell lines were analysed using a PCR-based methylation assay (Figure 2A). The DNA samples were precipitated with ethanol, dissolved in distilled water, and further digested with mCpG-sensitive *Bst*UI (1 *μ*g U^−1^, New England Biolabs, Beverly, MA, USA) at 60°C for 16 h. The *Bst*UI-digested DNAs then were amplified with specific primers for the *CKMT1* CpG island (forward: 5′-CAGTGGGTGTCTTTCCCAGT-3′; reverse: 3′-GTTCACTGGCAGCTCGTACA-5′). The PCR reactions were performed in a final volume of 25 *μ*1 containing 1 *μ*1 of digested DNA, 2.5 pmol of each specific primer, 50 *μ*M of dNTPs, 10 mM Tris-HCl buffer (pH 8.3), 50 mM KCl 1.5 mM MgCl_2_, and 0.5 U of AmpliTaq (Applied Biosystems, Foster City, CA, USA). The amplified PCR products were separated on 3% agarose gel and visualised by ethidium bromide after the run. DNA from the NOKs treated *in vitro* with *Sss* I methylase (New England Biolabs) was used as a positive control for methylated alleles. The *Sss* I-untreated DNA was used as a negative control for methylated genes. To assess reactivation of *CKMT1* mRNA expression, demethylating assay was performed using the DNA methyltransferase inhibitor, 5-aza-2′-deoxycytidine (5-aza-2′-dC) (Sigma Chemical Co.), as described previously (Yamamoto *et al*, 2001) (Figure 2A). The cells were treated with different concentrations (0 and 2 *μ*M) of 5-aza-2′-dC. On day 5, the cells were washed with PBS and grown for another 10 days without the demethylating chemical. The cells then were harvested, and total RNA was extracted, and the expression of the *CKMT1* gene was evaluated by real-time quantitative RT–PCR as described above.

### Transiently transfection

Human *CKMT1* cDNA was cloned into a pME18SFL3 expression vector for transient transfection experiments (Joshi *et al*, 2002) (Figure 2A). The nine OSCC-derived cell lines were transfected with pME18SFL3 encoding *CKMT1* cDNA using the nonliposomal formulation FuGENE-6 transfection reagent (Roche; Tao *et al*, 2004). All experiments using these cells were performed 48 h after transfection. Mock transfection of the nine OSCC-derived cell lines cultures with pME18SFL3 expression vector alone was used as a control. Real-time quantitative RT–PCR analyses and immunofluorescence were performed to confirm transfection efficiency.

### *In vitro* invasion assay

The invasive activity of the nine CKMT1-transfected cells and nine mock-transfected cells *in vitro* were tested using a QCM 96-Well Cell Invasion Assay Kit (Chemicon, Temecula, CA, USA; Wang *et al*, 1997; Ciolczyk-Wierzbicka *et al*, 2004) (Figure 2A). The cells were loaded into a chamber coated with ECMatrix at a density of 5 × 10^5^ cells ml^−1^ in 0.1 ml serum-free Dulbecco's modified Eagle's medium F-12 HAM. The plates with cells were incubated for 16 h at 37°C in a CO_2_ incubator. Migrating cells on the bottom of the insert were incubated with 150 *μ*1 of prewarmed cell detachment buffer for 30 min, dissociated from the membrane, and detected by CyQuant GR dye. The fluorescence plate was read using a 480/520 nm filter set.

### Apoptosis assay

Apoptosis detection was carried out using a terminal deoxynucleotidyl transferase-mediated deoxyuridine triphosphate (dUTP)-biotin nick end labeling (TUNEL) method (Tornusciolo *et al*, 2004) with an *In situ* Apoptosis Detection Kit (Takara), according to the manufacturer's protocol (Figure 2A). Briefly, the cells were fixed in 4% neutral buffered formalin, dried onto microscope slides, washed with PBS, equilibrated, and incubated with terminal deoxynucleotidyl transferase in a reaction buffer containing digoxigenin dUTP at 37°C for 1 h. The reaction was stopped, the specimens were washed for 30 min, and the slides were incubated with anti-digoxigenin antibody coupled to FITC for 30 min at room temperature and washed three times with PBS before mounting for photomicrography under phase and epifluorescence illumination. Apoptotic index was determined by calculating the percentage of cells that was apoptotic through positive staining. All slides were blindly evaluated by three independent times.

## RESULTS

### Two-dimensional PAGE

**Figure 1:**
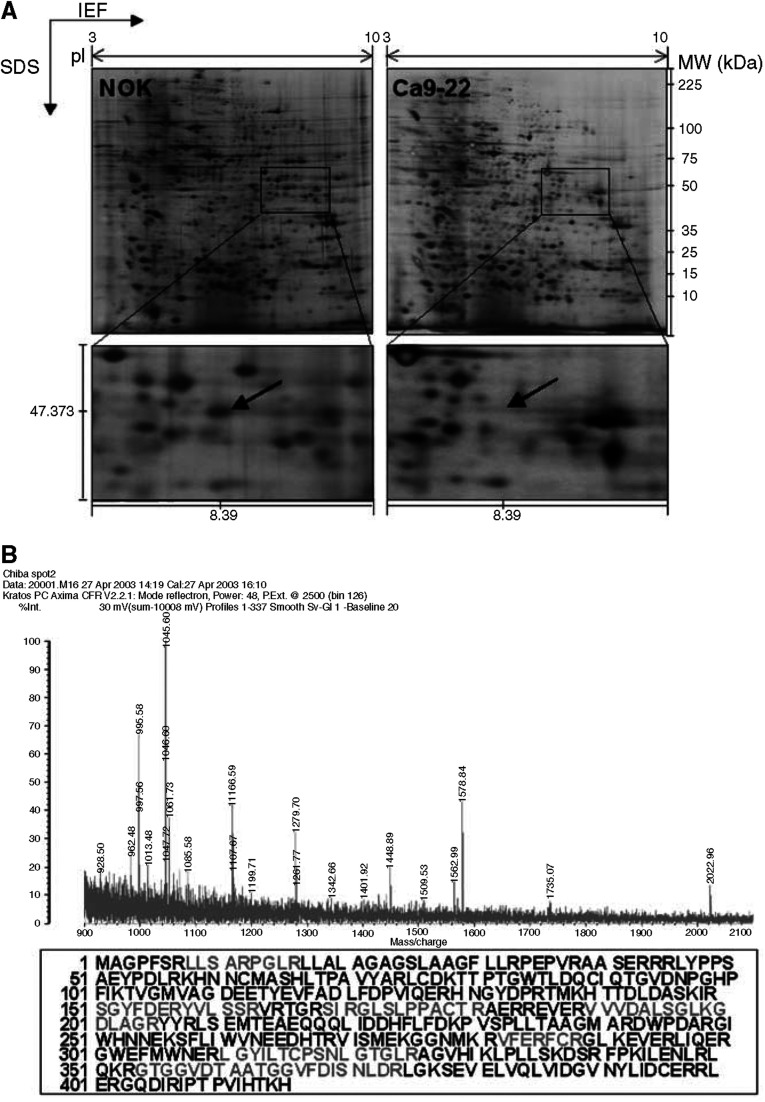
(**A**) Representative master 2-DE gel images of NOK and OSCC-derived cell line (Ca9-22) samples. After isoelectric focusing for the first dimension of protein separation, the gel strips were electrophoresed in vertical SDS–PAGE slab gels and stained with silver. Close-ups of 2-DE gel images were significantly downregulated in the OSCC-derived cell lines compared with the NOKs. Mw, molecular weight. (**B**) Results of MALDI-TOF-mass spectrometric analysis of this spot. Amino-acid sequences analysed by peptide mass fingerprinting analysis are redlined on the full-length sequence of the CKMT1 protein.

Protein samples were extracted from the OSCC-derived cell lines and concentrations determined by the Bradford method using SmartSpec 3000 (Bio-RAD, Hercules, CA, USA). Total protein extraction rates did not differ significantly between the OSCC-derived cell lines and NOKs. Usually 100 *μ*g of proteins per sample were used for 2-D PAGE separation. The proteins were distributed evenly in the 7-cm gel with isoelectric points in the range of 3–10 and molecular masses of 6–200 kDa. Figure 1A shows typical master gel images for OSCC-derived cell lines and NOK samples. About 450 protein spots in each gel were detected using the Phoretix Two-Dimensional Advanced v5.01 analysis program. Proteins with a >2-fold change in expression were considered significant. Three proteins were upregulated and 27 proteins were suppressed significantly in OSCC-derived cell lines compared with NOKs.

### Protein identification by peptide-mass fingerprinting

Each protein spot was excised and subjected to in-gel tryptic digestion, MALDI-TOF-mass measurement, and database matching. We identified five candidate proteins that were differentially expressed in OSCC cell lines compared with NOKs. Table 1 shows the proteins identified; most of the matched proteins had high sequence coverage, mass accuracy, and MOWSE scores. We identified CKMT1 as a potentially novel molecular marker for OSCCs using this technique (Figure 1B).

### Immunofluorescence

**Figure 2:**
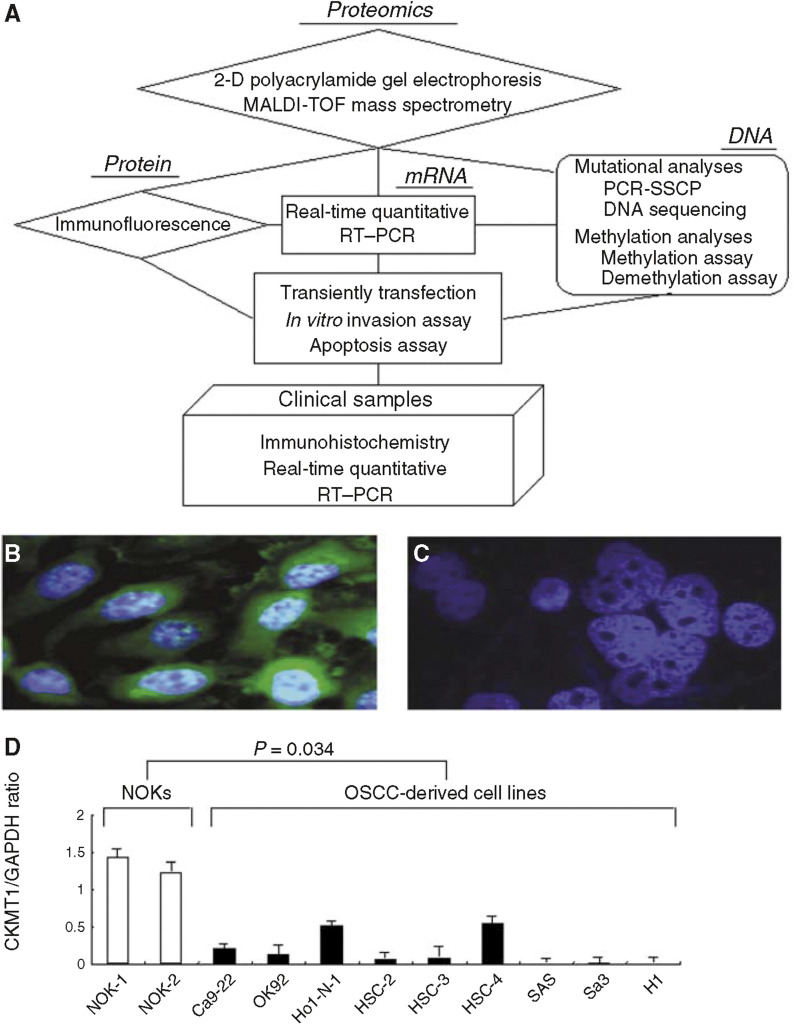
(**A**) A diagram of the experimental process. For details, see Materials and Methods. Immunofluorescence analyses show the status of CKMT1 protein expression. The CKMT1 proteins of the NOK (**B**) are surrounded by green fluorescence; fluorescence is not seen in the OSCC-derived cell line (OK92) samples (**C**). (**D**) Quantification of mRNA levels in OSCC-derived cell lines by real-time RT–PCR analysis. Significant downregulation of the *CKMT1* gene is seen in all cell lines compared to *CKMT1* mRNA expression in NOKs. Data are expressed as means±s.d.

We examined the expression level of the CKMT1 proteins based on the results of proteomic analyses by immunofluorescence analysis. Nine OSCCs-derived cell lines and NOKs were examined. The CKMT1 proteins in the NOKs were surrounded by obvious green fluorescence; no fluorescence was seen in all OSCC-derived cell lines. The data were matched to the proteomic study. Figure 2C shows a representative result of significant decrease expression of CKMT1 protein in the OSCC-derived cell line, when compared to its expression in NOK (Figure 2B).

### Immunohistochemistry

**Figure 3:**
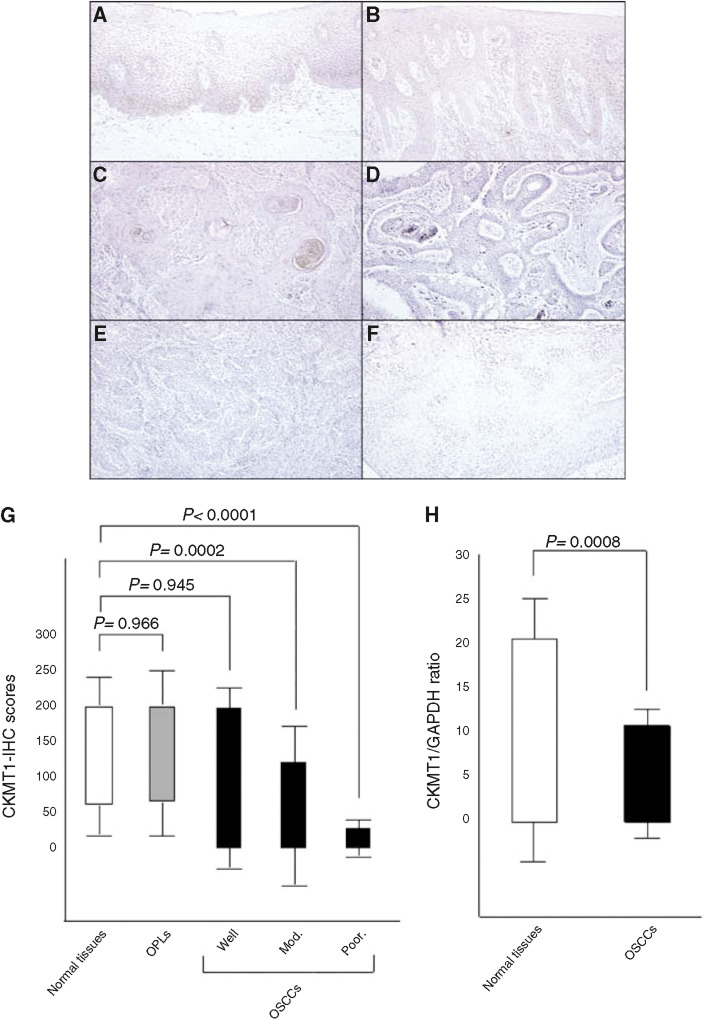
Immunohistochemistry staining of CKMT1 in normal and tumorous oral tissues. (**A**) Normal oral tissue exhibits CKMT1 protein expression, which is limited to the cytoplasm of the epithelial cells. (**B**) CKMT1-positive case of OPL (leukoplakia). (**C**) CKMT1-positive case of well-differentiated primary OSCC. (**D**) CKMT1-negative case of well-differentiated primary OSCC. (**E**) CKMT1-negative case of moderately differentiated primary OSCC. (**F**) CKMT1-negative case of poorly differentiated primary OSCC. Original magnification × 200. (**G**) CKMT1 protein expression levels in moderately differentiated and poorly differentiated tumours are significantly lower than in normal oral tissues. No significant difference in the CKMT1 IHC scores is seen among normal oral tissue, OPLs, and well-differentiated tumours. (**H**) *CKMT1* mRNA expression status in primary OSCCs. The relative mRNA expression level in normal tissues and primary OSCCs ranges from 0.1 to 20.1 (mean, 2.02) and 0.02 to 10.6 (mean, 0.86), respectively. There is a significant difference in the CKMT1 mRNA expression levels between negative and positive cases (*P*=0.0008, Mann–Whitney's *U*-test).

Among 52 OSCCs analysed by IHC staining, 19 had significantly decreased expression of CKMT1 (IHC score <62.2). In contrast, all normal tissues had a strong cytoplasmic immunoreaction for CKMT1. There was a statistically significant difference between the frequency of CKMT1-negative cases and clinicopathologic features (Table 2). All 21 (100%) oral premalignant lesions (OPLs) were CKMT1-positive. Figure 3A–F shows representative results for CKMT1 protein expression in normal oral tissue, OPLs, and primary OSCCs. The CKMT1-IHC scores for normal tissues, OPLs, OSCCs, well, moderately and poorly differentiated type of OSCCs, respectively, ranged from 62.2 to 200 (mean, 116.5), 67.5 to 200 (mean, 120), 0 to 200 (mean, 88.5), 0 to 200 (mean, 92.6), 0 to 123.4 (mean, 56.6), and 0 to 29.6 (mean, 12.6). CKMT1 expression levels in both moderately and poorly differentiated tissues were significantly lower than those in normal oral tissues (*P*=0.0002 and *P*=0.0001, respectively). In contrast, we found no significant difference in the CKMT1 IHC scores among normal oral tissue, OPLs, and well-differentiated tumours (*P*=0.966 and *P*=0.945, respectively) (Figure 3G).

### mRNA expression analysis

Real-time quantitative RT–PCR analysis data were matched to the protein expression values in the 2-DE analyses, immunofluorescence analyses, and immunohistochemistry analyses. mRNA expression of CKMT1 was significantly reduced in tumours of randomly selected CKMT1-negative cases (*n*=21) compared to the selected CKMT1-positive cases (*n*=29). mRNA expression levels were normalised to GAPDH. The relative mRNA expression level in the normal tissues and primary OSCCs ranged from 0.1 to 20.1 (mean, 2.02) and 0.02 to 10.6 (mean, 0.86), respectively (Figure 3H). There was no statistically significant difference between the frequency of CKMT1-negative cases and clinicopathologic features (Table 2). A significant decrease in CKMT1 expression was observed in OSCC-derived cell lines compared with NOKs used as controls (Figure 2D). The data are expressed as the mean±s.d. of three independent experiments with samples in triplicate.

### Mutational analyses

To investigate the mechanisms responsible for downregulation of *CKMT1* gene expression in OSCC-derived cell lines, we analysed genomic DNA by PCR-SSCP and direct sequence analyses. A band shift was detected in four of the nine (44%) OSCC-derived cell lines (Ho-1-N-1, HSC-2, HSC-4, SAS). Sequence variations were observed in exon 8. All sequencing variations were nonsense mutations (AAA changed to AAG and lysine changed to lysine) at codon 407 (data not shown).

### Methylation analyses

**Figure 4:**
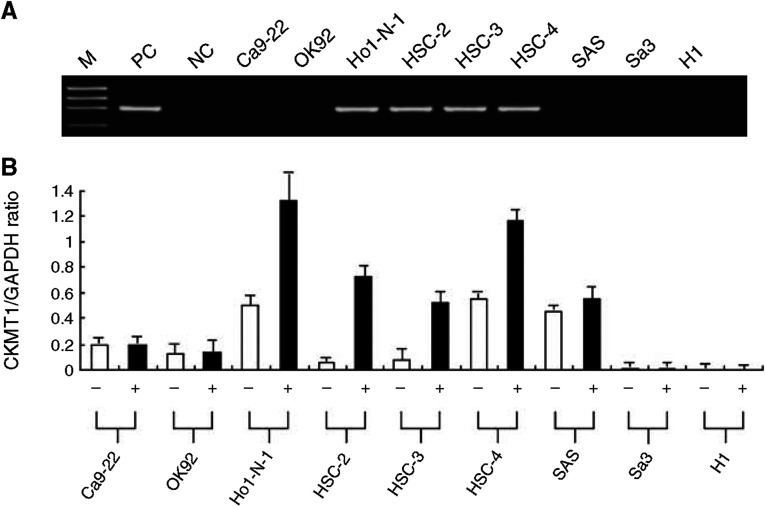
(**A**) Typical results of the methylation assay. CKMT1 methylation is seen in four OSCC cell lines (Ho-1-N-1, HSC-2, HSC-3, and HSC-4), which show reduced *CKMT1* mRNA expression. DNA from NOK treated *in vitro* with *Sss*I methylase is used as a positive control (lane PC) for methylated alleles. *Sss*I-untreated DNA is used as a negative control (lane NC) for methylated genes. M, molecular marker. (**B**) Quantitative real-time RT–PCR analysis for demethylation assay in *CKMT1*-methylated OSCC cell lines. Restoration or upregulation of *CKMT1* mRNA expression is detected in all *CKMT1*-methylated OSCC cell lines after treatment with the DNA demethylating agent 5-aza-2′-dC. +, 2 *μ*M 5-aza-2′-dC treatment; −, 0 *μ*M 5-aza-2′-dC treatment.

We analysed the methylation status of the CpG island region of the *CKMT1* gene in the 9 OSCC-derived cell lines. CKMT1 methylation was detected in four (44%) of the cell lines (Ho-1-N-1, HSC-2, HSC-3, HSC-4), whereas NOKs had no methylation allele. Figure 4A shows representative results of methylation status. To further study the consequences of loss of expression of CKMT1 in association with CpG island hypermethylation in OSCC-derived cell lines, the cell lines with methylation and transcriptional inactivation of CKMT1 were treated with 5-aza-2′-dC. Significant upregulation (in Ho-1-N-1, HSC-2, HSC-3, HSC-4) of mRNA expression was observed after 5-aza-2′-dC treatment (Figure 4B). The results were matched to methylation states studied in a PCR-based methylation assay. The data are expressed as the mean±s.d. of three independent experiments with samples in triplicate.

### Functional analysis of CKMT1

**Figure 5:**
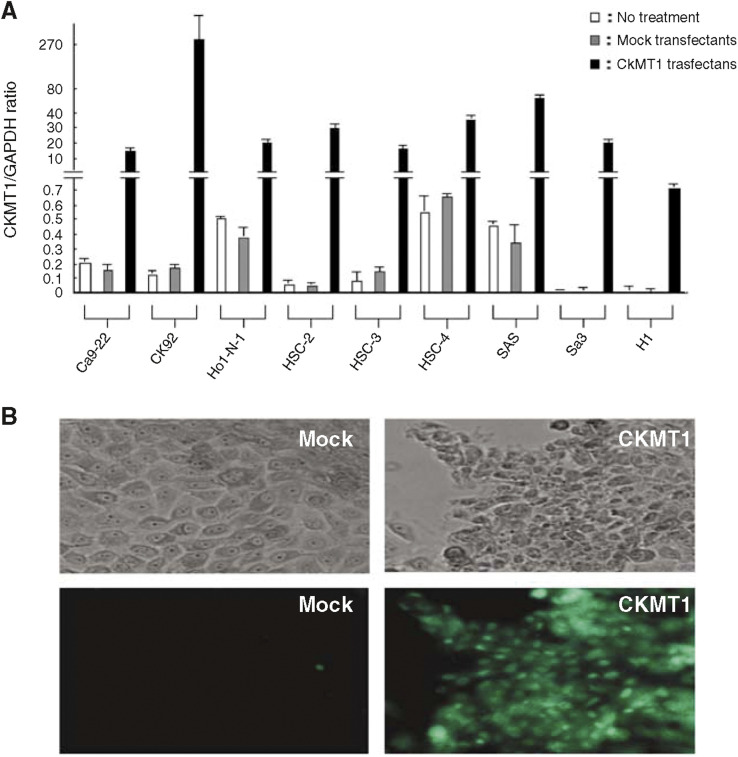
CKMT1 transiently transfected efficiency is confirmed by quantitative real-time RT–PCR analyses and immunofluorescence. (**A**) Quantitative real-time RT–PCR revealed that CKMT1 is overexpressed 30–5000-fold in all CKMT1 transfectants compared with mock transfectants and untreated cell lines. (**B**) Representative result of TUNEL assay. More apoptotic cells were clearly seen in HSC-2-CKMT1 transfectants than in mock transfectants.

To confirm transfection efficiency, quantitative real-time RT–PCR analyses and immunofluorescence were carried out. Quantitative real-time RT–PCR showed that CKMT1 was overexpressed 30–5000-fold in CKMT1-transfected OSCC-derived cell lines compared with mock-transfected OSCC-derived cell lines and untreated cell lines (Figure 5A). On immunofluorescence, no significant difference in the CKMT1 expression pattern was observed between mock-transfected OSCC-derived cell lines and untreated cell lines. In contrast, all CKMT1-transfected OSCC-derived cell lines had a strong immunoreaction for CKMT1.

The invasive activity of the nine CKMT1-transfected cell lines and the nine mock-transfected cell lines were tested using the ECMatrix invasion chamber assay. Transfection of OSCC-derived cell lines with *CKMT1* produces no significant decrease in invasive activity (data not shown).

To assess the specific role of CKMT1 downregulation, we evaluated the apoptotic ability between CKMT1-transfected OSCC-derived cell lines and the mock-transfected OSCC-derived cell lines. Cell morphology was analysed using optical microscopy. Morphological change showing apoptotic bodies, such as decrease of cellular and nuclear sizes, were observed in all CKMT1-transfected OSCC-derived cell lines, but not in mock-transfected OSCC-derived cell lines. TUNEL-positive cells were virtually undetected in mock-transfected OSCC-derived cell lines, whereas all the CKMT1-transfected OSCC-derived cell lines had increased numbers of TUNEL-positive cells (Figure 5B). The apoptotic index for CKMT1-transfected OSCC-derived cell lines and mock-transfected OSCC-derived cell lines, respectively, ranged from 26.92 to 76.58 (mean, 43.25), 2.77 to 14.12 (mean, 6.42).

## DISCUSSION

We identified CKMT1 as a potentially novel molecular marker for OSCCs. Creatine kinase (CK), originally found in cytosol and the mitochondria of cells, catalyses the reversible exchange of high-energy phosphate between ATP and phosphocreatine (PCr; Wallimann *et al*, 1992; Wallimann *et al*, 1998). Cytosolic CKs are dimmers consisting of muscle-type subunits, brain-type subunits, or both. However, two genotypes of mitochondrial CK have been identified, that is, CKMT1 and the sarcomeric mitochondrial CK that are encoded by two different genes on human chromosomes 15 and 5 and expressed in a tissue-specific manner (Stallings *et al*, 1988; Haas *et al*, 1989). CKMT1 is coexpressed with the cytosolic brain-type subunits in many cells and tissues with high-energy demand, such as the brain, placenta, kidney, testis, sperm, or endothelial cells. The enzyme is thought to have two main functions in energy metabolism (Wallimann *et al*, 1992): it buffers the cellular ATP pool by maintaining high cytosolic concentrations of PCr, which can be used in times of high cellular energy demand to regenerate ATP, and it maintains an energy shuttle between subcellular sites of energy supply (oxidative phosphorylation, glycolysis) and sites of energy demand using the easily diffusible creatine (Cr)/PCr. For example, CKMT1, located in the mitochondrial intermembrane space, preferentially catalyses the phosphorylation of Cr to PCr, which then diffuses into the cytosol where it is used in a reverse reaction by cytosolic CK to restore ATP for different, often closely associated ATPases (Schlattner *et al*, 1998). In addition, CKMT1 has been implicated in the regulation of the Ca^2+^-induced mitochondrial permeability transition pore (PTP; O'Gorman *et al*, 1997). PTP is involved in triggering apoptosis by releasing proapoptotic factors into the cytosol (Crompton, 1999). Several studies have shown the aberrant expression of CKMT1 in cancers of various organs, including lung cancer, breast cancer, gastric cancer, colon cancer, and prostate cancer (DeLuca *et al*, 1981; Tsung, 1983; Kanemitsu *et al*, 1984; Wallimann *et al*, 1992), suggesting that CKMT1 is involved in the pathogenesis of various cancers. However, there has been no report regarding CKMT1 expression in oral cancer, which lead us to further analyse this potential protein.

In the present study, we showed the state of *CKMT1* gene mutation and its mRNA/protein expression in OSCC-derived cell lines and NOKs by SSCP/DNA sequencing, quantitative real-time RT–PCR, and immunofluorescence. Our data indicate that CKMT1 expression decreases or is lost in OSCC-derived cell lines but is abundant in NOKs. When DNA from OSCC-derived cell lines was analysed, we found nonsense mutations in the coding sequence of the *CKMT1* gene. In contrast to mutational analyses, quantitative real-time RT–PCR analysis of *CKMT1* mRNA showed frequent downregulation of the gene in all cell lines, indicating that other mechanisms, such as post-transcriptional modification and upregulated degradation, may be involved in *CKMT1* gene silencing. In this context, with accumulating knowledge of the mechanisms of inactivation of tumour-suppressor genes, abnormal methylation at the promoters of tumour-suppressor genes is another mechanism that suppresses gene activity (Jones and Laird, 1999). In oral tumours, the promoters of several tumour-suppressor genes, that is, p16, p15, p14, and E-cadherin, are highly methylated in addition to a rare gene mutation in human OSCCs (Saito *et al*, 1998; Miracca *et al*, 1999; Shintani *et al*, 2001). In the present study, we found an association between methylation and suppression of *CKMT1* gene expression in OSCC-derived cell lines. Treatment of OSCC-derived cell lines showed that *CKMT1* methylation with a demethylating agent restored or significantly upregulated *CKMT1* expression. These findings indicate that reduced expression of the *CKMT1* gene may be linked to human oral carcinogenesis and/or progression of cancer due to epigenetic silencing. Thus, we suggest that CKMT1 could be a class II tumour-suppressor gene in that it is structurally intact in sequence but underexpressed or unexpressed due to downregulation or silencing in transcription or translation (Jones and Laird, 1999). These data show that proteomic analyses coupled with genetic and epigenetic analyses may be an essential strategy to discover novel molecular targets in the development of OSCCs.

To confirm the correlation between invasiveness and reduction of the CKMT1 protein, we examined the invasive activity of CKMT1-transfected OSCC-derived cell lines and mock-transfected OSCC-derived cell lines by *in vitro* invasion assay. The results showed that transfection of OSCC-derived cell lines with *CKMT1* produces no substantial difference in invasive activity. We suggest that CKMT1 is unrelated to the invasiveness of the cells and suppresses or promotes metastasis of OSCCs.

To assess the specific role of CKMT1 downregulation, we evaluated apoptotic ability. The results showed that all CKMT1-transfected OSCC-derived cell lines had increased TUNEL-positive cells compared with mock-transfected OSCC-derived cell lines. Apoptosis can be mediated via several pathways, one of which involves the engagement of the so-called death receptors belonging to the tumour necrosis factor receptor superfamily. A cascade of proteolytic digestion occurs, involving caspases, resulting in cell death. However, caspase-8 may digest the Bid protein to yield a truncated form that can induce mitochondrial damage, eventually leading to cell death. In addition, many agents induce cellular stress, which can also lead to mitochondrial perturbation and ultimately cell death. A mechanism causing the mitochondrial dysfunction mentioned previously has been proposed that consists of mitochondrial membrane permeability transition, dissipation of the inner membrane potential, osmotic swelling of the matrix, rupture of the outer mitochondrial membrane, release of cytochrome *c* and other apoptogenic proteins from the mitochondria, and formation of the caspase-3 activation complex, the apoptosome (Debatin *et al*, 2002; Newmeyer and Ferguson-Miller, 2003). Permeability transition involves the opening of a PTP. The main components of this pore are adenine nucleotide translocator (ANT) and cyclophilin D in the inner membrane of the mitochondria and a voltage-dependent anion channel and a peripheral benzodiazepine receptor (VDAC) in the outer mitochondrial membrane. The PTP is formed in regions of contact between the inner and outer mitochondrial membranes. Prolonged opening of the PTP leads to the previously mentioned effects, exposes cytosol to the contents of the mitochondria, and culminates in cell death (Debatin *et al*, 2002; Newmeyer and Ferguson-Miller, 2003). Interestingly, mitochondrial permeability transition can lead to both apoptosis and necrosis (Lemasters *et al*, 1998). CKMT1 is located in the intermembrane space and also interacts with VDAC–ANT complexes (Marzo *et al*, 1998). We suggest that CKMT1 might induce apoptosis through specialised systems such as the PTP in OSCCs.

We showed the status of *CKMT1* expression in clinical tissue samples obtained from primary OSCCs and the corresponding normal oral mucosa by quantitative real-time RT–PCR and IHC staining; high frequencies of CKMT1 downregulation were detected. Moreover, no difference in protein expression was observed in OPLs. In addition, we found a significant correlation between *CKMT1* expression status and the clinicopathologic features. Most of the primary OSCCs diagnosed as moderately or poorly differentiated tumours showed downregulation of CKMT1 protein expression, and a statistically significant difference was observed between the CKMT1-reduced primary OSCCs and the histopathologic type (*P*<0.0001). Recently, a relation between mitochondrial homeostasis and tumour differentiation was reported (Krueger, 1995; Morgan *et al*, 2004). For example, the VDAC is mainly found on the outer mitochondrial membrane of cells as described previously. The VDAC plays a role in several cellular functions including haem synthesis, steroidogenesis, DNA synthesis, cell growth and differentiation, and apoptosis (Krueger, 1995). In cutaneous neoplasms and other skin diseases, a heterogeneous pattern of VDAC expression at lower intensity was seen depending on the tumour type and the degree of differentiation (Morgan *et al*, 2004). The VDAC expression was greatest in well-differentiated tumours, synonymous with the VDAC expression gradient seen in normal skin, and least in poorly differentiated and infiltrative tumours (Morgan *et al*, 2004). Although we have not examined the relationship between CKMT1 expression and oral cancer cell differentiation *in vitro*, above-mentioned evidences and our data suggest that change of mitochondrial homeostasis, in part, causes tumour differentiation. Furthermore, the potential role of *CKMT1* in the regulation of both differentiation and apoptosis could be involved in the development of OSCC.

Based upon our data, we have concluded that downregulation of CKMT1 is, in part, involved in the oral carcinogenesis and that an epigenetic mechanism may regulate loss of its expression, which leads to block apoptosis through specialised systems such as mitochondrial permeability transition pores in oral cancer cells. Further investigations with greater number of clinical samples including precancerous lesions are needed to improve our ability to diagnose, prevent, and treat this neoplasm.

## Figures and Tables

**Table 1:** Results of MALDI-TOF mass spectra and database searching for protein identification

**Protein name**	**Peptides matched**	**Sequence coverage**	**Mowse score^a^**	**Cancer cells (up or down)**
Creatine kinase, mitochondrial	8	20(%)	97	Down
Acetyl-Coenzyme A acyltransferase	9	28(%)	92	Down
Aldolase C, fructose-bisphosphate	9	23(%)	86	Up
Similar to hydroxyacyl-Coenzyme A dehydrogenase	8	37(%)	106	Up
Dystrophin-related protein 3	8	19(%)	70	Up

a MOWSE scores over 67 were significant (*P*<0.05).

MALDI-TOF-mass=matrix-assisted laser desorption/ionisation time of fly mass spectrometry.

**Table 2:** Correlation between CKMT1expression and classification in oral squamous cell carcinomas

	**Result of immunostaining: no. of patients (%)**					**Result of real-time PCR: no. of patients (%)**		
**Clinical classification**	**Total**	**CKMT1 (−)**	**CKMT1 (+)**	***P*-value**	**Total**	**CKMT1 (−)**	**CKMT1 (+)**	***P*-value**
*Age at surgery (year)*								
⩽49	12	2(17)	10(83)		6	2(33)	4(67)	
50–59	16	9(56)	7(44)	0.208971	13	6(46)	7(54)	0.722219
60–69	12	4(33)	8(67)		13	7(54)	6(46)	
70	12	4(33)	8(67)		18	6(33)	12(67)	
								
*Gender*								
Male	31	13(42)	18(48)		32	13(41)	19(59)	1
Female	21	6(29)	15(71)	0.388742	18	8(44)	10(56)	
								
*T-primary tumour*								
T1	4	0(0)	4(100)		5	2(40)	3(60)	
T2	26	9(35)	17(65)	0.223836	30	9(30)	21(70)	0.117337
T3	6	4(67)	2(33)		5	3(60)	2(40)	
T4	16	6(38)	10(62)		10	7(70)	3(30)	
								
*N-regional lymph node*								
N(−)	38	15(39)	23(61)	0.533534	18	5(28)	13(72)	0.1485
N(+)	14	4(29)	10(71)		32	16(50)	16(50)	
								
*Stage*								
I	4	0(0)	4(100)		2	1(50)	1(50)	
II	15	5(33)	10(67)	0.115748	13	3(23)	10(77)	0.213194
III	5	4(80)	1(20)		14	4(29)	9(71)	
IV	28	10(36)	18(64)		21	12(57)	9(43)	
								
*Histopathologic type*								
Well differentiated	35	5(14)	30(86)		42	15(36)	27(64)	
Modelately differentiated	11	8(73)	3(27)	<0.0001	7	5(71)	2(29)	0.0633215
Poorly differentiated	6	6(100)	0(0)		1	1(100)	0(0)	
								
*Tumour site*								
Gingiva	16	5(31)	11(69)		17	7(41)	10(59)	
Tongue	26	10(38)	16(62)		19	6(32)	13(68)	
Buccal mucosa	4	0(0)	4(100)	0.298623	7	3(43)	4(57)	0.30294
Oral floor	3	2(67)	1(33)		2	1(50)	1(50)	
Oropharyngeal isthmus	3	2(67)	1(33)		2	2(100)	0(0)	
Lower lip	—	—	—		2	2(100)	0(0)	
Soft palate	—	—	—		1	0(0)	1(100)	
Leukoplakias	21	0	21	—	—	—	—	—

**Table 3:** Primers and parameters for PCR-single-strand confirmation polymorphism (SSCP) analysis

**Gene**	**Forward (5′–3′)**	**Reverse (3′–5′)**	**Product size (bp)**	**Number of cycle**	**Annealing condition**
*CKMT1 (15q15)*					
Exon1	CCCTGTTCCGGATCTTATCT	CCGGATTCTCACTTCTACCC	292	35	64°C, 10 s
Exon2	CCCTCCATGGTTACTGGGTA	TGGATCATCAGGGAGACTCTG	264	35	64°C, 10 s
Exon3	TTGAAGTCTGGGGAGGGATT	ACACAGGCAATGCAAATCAG	196	35	64°C, 10 s
Exon4	CAATGCATGCAGGAAGAATG	CTGCTGTTCAGCCTCTGTCA	332	35	64°C, 10 s
Exon5	CCAGATGAGACATGGGCTCT	TCAAGAGGCAAGGCAAGAAT	254	35	64°C, 10 s
Exon6	ATTCTTGCCTTGCCTCTTGA	ATGTCCTGGGTTGGTTGGTA	267	35	64°C, 10 s
Exon7	TAAGGGAGGGTCCAGGGTTA	CTTTCCAAAGGGTCCACTCC	283	35	64°C, 10 s
Exon8	CAGGTTCAGGGCTCTTTCAG	CTTGCCTGCTCATTTCCAAT	344	35	64°C, 10 s
Exon9	ATTGGAAATGAGCAGGCAAG	AGACAGCTGCAGCATAGCAA	343	35	64°C, 10 s
